# Efficacy and Safety of a Bovine-Associated *Staphylococcus aureus* Phage Cocktail in a Murine Model of Mastitis

**DOI:** 10.3389/fmicb.2017.02348

**Published:** 2017-11-28

**Authors:** Koen Breyne, Ryan W. Honaker, Zachary Hobbs, Manuela Richter, Maciej Żaczek, Taylor Spangler, Jonas Steenbrugge, Rebecca Lu, Anika Kinkhabwala, Bruno Marchon, Evelyne Meyer, Lucia Mokres

**Affiliations:** ^1^Department of Pharmacology, Toxicology and Biochemistry, Faculty of Veterinary Medicine, Ghent University, Ghent, Belgium; ^2^EpiBiome, Inc., San Francisco, CA, United States; ^3^VDx Veterinary Diagnostics and Preclinical Research Services, Davis, CA, United States

**Keywords:** antibiotic alternatives, phage therapy, bovine mastitis, *S. aureus*, mouse model

## Abstract

Overuse of antibiotics is a major problem in the treatment of bovine mastitis, and antibiotic treatment is frequently non-curative, thus alternative treatments are necessary. The primary aim of this study was to evaluate the efficacy of a purified phage cocktail for treatment of bovine *Staphylococcus aureus* mastitis in a well-defined mouse model. Candidate phages were selected based on their *in vitro* performance and subsequently processed into an optimally composed phage cocktail. The highest scoring phages were further tested for efficacy and resistance suppression in broth and raw milk, with and without supplemental IgG. As these *in vitro* results displayed significant decreases in CFU, the cocktail was purified for testing *in vivo*. Lactating mice were intramammarily inoculated with *S. aureus* N305 (ATCC 29740), a clinical bovine mastitis isolate commonly used for experimental infection of dairy cows. The phage cocktail was applied via the same route 4 h post-inoculation. Treated mammary glands were graded for gross pathological appearance and excised for bacterial and phage load quantification as well as histopathology. Observation of gross macroscopic and histopathological changes and CFU quantification demonstrated that the phage cocktail treatment significantly improved mastitis pathology and decreased bacterial counts. Phage PFU quantification indicated that the tested phage cocktail treatment was able to maintain high intramammary phage titers without spreading systemically. The *in vivo* results complement the *in vitro* data and support our concept of phage therapy as an innovative alternative or supplementation therapy to antibiotics for the treatment of bovine mastitis.

## Introduction

Bovine mastitis is the most prevalent disease impacting United States dairy cattle (USDA, 2007). Mastitis is defined as inflammation of the udder, commonly caused by bacterial infection via the teat canal. Culling rates due to mastitis are typically around 15% of dairy cows in a herd (Jones, 2009). Although several bacterial species can cause mastitis, one of the most problematic pathogens is *Staphylococcus aureus*, a Gram-positive pathogen that infects between 3 and 15% of dairy cows in a herd (Jones, 2009).

Small-molecule antibiotic therapy is currently used to both treat and prevent *S. aureus* mastitis (Bradley and Green, 2001; Roy and Keefe, 2012; Gomes and Henriques, 2016). Treatment of clinical mastitis typically occurs during the lactation cycle and involves selective intramammary (IMM) infusion of antibiotics into the infected udder quarters. In contrast, during the dry period (i.e., the period of non-lactation prior to the next parturition), antibiotics may be administered into all quarters regardless of their infection status to treat subclinical, undetected mastitis and prevent the emergence of mastitis both during the dry period and at the time of parturition. Antibiotic treatment success rates are highly variable. Despite the antibiotic sensitivity that most *S. aureus* mastitis isolates display *in vitro* (Erskine et al., 2006; Lindeman et al., 2013), they are more difficult to eliminate *in vivo* with reported clinical mastitis cure rates as low as 4% (Barkema et al., 2006). Although milk is rigorously tested for antibiotic residues, consumers have nevertheless become more concerned about antibiotics in the food supply (Lusk et al., 2006). The perception of overuse of antibiotics in human and veterinary medicine has also caused concern, motivating the United States government in 2015 to release the National Action Plan for Combating Antibiotic-Resistant Bacteria (House, 2015). Thus, there is an unmet need to discover and develop alternative treatments for mastitis that do not rely on small-molecule antibiotics. Phage-based treatments offer promising potential as a therapeutic and/or preventative alternative to antibiotics due to their host specificity, thereby preserving the healthy microbiome, and their potential for avoiding antibiotic residues in milk. A wide variety of publications in the agricultural realm support their use, (example reviews Johnson et al., 2008; Atterbury, 2009). Currently, a withholding period is imposed after completing a course of antibiotic therapy in dairy cattle to allow the animal to eliminate the antibiotic from its system prior to returning to production for human consumption. The length of this period is determined by route of administration and pharmacokinetic properties of the specific drug, and residue studies are performed prior to product approval to determine when milk and meat from the cows is acceptable for consumption. During the withholding period, the antibiotic-contaminated milk must be discarded and the animal cannot be culled for the purpose of meat consumption. Milk discarded comprises one of the major negative economic impacts of mastitis (Halasa et al., 2007). If phage therapy were to be granted a zero withholding period based on a positive human food safety profile, the presence of any residual phage in meat or milk would be of no concern, it would benefit producers by reducing losses due to discarded milk and if appropriate, send cattle to slaughter sooner.

The primary aim of this pilot study was to evaluate for the first time the efficacy of a *S. aureus* phage cocktail containing an equal mixture of two selected top candidates using a validated murine model for bovine mastitis. This preclinical model has been established as an effective screening tool for potential bovine mastitis therapies (Brouillette et al., 2004; Brouillette and Malouin, 2005; Notebaert and Meyer, 2006). The phage cocktail efficacy was compared to the efficacy of a commercial antibiotic (used as positive control). Safety served as a secondary aim and was evaluated in an uninfected phage-only treated group of mice. The obtained results indicate a significant decrease in bacterial counts, decreased gross pathological signs and a lack of pathological response to this *in vitro* optimized phage cocktail, providing preclinical evidence for the success of phage therapy for treatment of bovine mastitis.

## Materials and Methods

### Phage Cocktail Formulation

Each phage within a panel of 12 potential cocktail candidates was tested using quantitative measures of infection including efficacy and kinetics of killing, amplifiability, time to resistance emergence, genomic relatedness, and phage–phage interaction (data not shown). Phages were characterized singly and as cocktails to determine the most optimal cocktail composition. The two highest-scoring phages across these categories were selected for the therapeutic cocktail used in this study. The first of the two phages included in the final cocktail was derived from an in-house directed evolution protocol from parent Myovirus phage ATCC 23361. The second phage, BP39, was generously provided by PhageLux (GenBank accession number NC_031046).

### *In Vitro* Testing

*Staphylococcus aureus* N305 was selected for use in this study. This strain is a bovine mastitis isolate that has been widely used for experimental infection of dairy cows and mice (Breyne et al., 2014; Peton et al., 2014). The phage cocktail was tested in tryptic soy broth (TSB, Becton Dickinson) and raw milk (Organic Pastures) with the addition of bovine IgG (Equitech-Bio) as a potential inhibitor of phage activity as previously reported (O’Flaherty et al., 2005a; Gill et al., 2006b; Tanji et al., 2015). The reported IgG concentration in milk varies between studies, however, for healthy milk it has been reported to be 0.45–1.34 mg/mL and for mastitic milk, 3–15 mg/ml (Tomassetti et al., 2013). In-house testing of a variety of store-purchased, raw, and mastitic milk samples (Bovine IgG ELISA Kit from Alpha Diagnostics, data not shown) revealed a broad range as well. Thus 5 and 20 mg/mL were chosen as a low and high representative concentration.

Culture conditions were designed to closely mimic those present in the mouse model. Bacterial cultures used in the experiment were prepared from exponential phase cultures [Optical density at 600 nm (OD_600 nm_) = 0.3-0.6], and diluted to 2.5 × 10^3^ CFU/mL in a final volume of 200 μl of TSB or milk. These were shaken at 250 rpm horizontally in Eppendorf tubes at 37°C with supplemental IgG at 5 and 20 mg/mL for 4 h prior to the addition of phage. Prior to the experiment raw milk was tested on agar plates selective for *S. aureus* (CSA, CHROMagar) to verify that contaminating *S. aureus* were not present. IgG solutions were prepared by suspending lyophilized IgG in reagent grade water to a final concentration of 100 mg/mL, then filtering it through a 0.2 μm PES filter into a sterile Falcon tube. Both *S. aureus* phages from the selected phage cocktail were combined by diluting in sterile phosphate buffer saline (PBS) to 5 × 10^9^ PFU/mL per phage (1 × 10^10^ PFU/mL total). After 4 h of pre-treatment incubation, 200 μl of this phage cocktail was added to each Eppendorf tube to a final volume of a 400 μl and returned to 37°C shaking as before. After 3 and 18 h of incubation bacterial growth was assessed by taking 15 μl from each sample and performing serial dilutions in PBS using 96-well microplates. Next, 3 μl of each dilution was spotted on a CSA plate and incubated at 37°C overnight. On the next day pink/mauve *S. aureus* colonies were counted and CFU/mL was calculated for each sample.

### Phage Cocktail Amplification and Purification

Phage stocks were amplified on *S. aureus* N305 growing in TSB (Difco TSB powder dissolved in endotoxin-free water, used throughout to minimize endotoxin input) in Fernbach flasks using amplification and lysing conditions similar to (Bryan et al., 2016). Briefly, 1 L cultures were grown to OD_600 nm_ ∼ 0.2 (∼4 × 10^7^ CFU) and were infected at a multiplicity of infection (MOI) of 0.1 and incubated at 37°C for 4 h with shaking at 250 rpm before lysing with 2 mL of CHCl_3_. The resulting phage lysates were incubated overnight statically at room temperature in the dark. The following day, cultures were separated from any remaining chloroform and centrifuged for 20 min at 5353 × *g* to pellet bacterial debris, after which the supernatant was sterilized by membrane filtration through a 0.2 μm polyethersulfone (PES) filter. The resulting solution was concentrated using tangential flow filtration and the retentate product was then again sterilized by filtration through a 0.2 μm PES filter. Concentrated stocks were purified using anion exchange chromatography (Adriaenssens et al., 2012) with separate strong anion exchanger columns for each phage with a subset of the elutions containing the highest phage titers being buffer exchanged either using a size exclusion column or tangential flow filtration system into Ringer’s solution (116 mM NaCl, 2.9 mM KCl, 1.8 mM CaCl_2_^∗^H_2_O, 5 mM HEPES Buffer). Buffers used for all three systems were filtered-sterilized and prepared using endotoxin-free water.

Prior to final cocktail formulation and treatment, the purity of each phage solution was assayed by measuring the following parameters: phage titer was measured using a standard double agar plating method; bacterial DNA levels were quantified by qPCR-amplifying 16S rRNA (the calculated concentration of bacterial DNA took into account the average of five 16S copies and 2,782,562 base pairs present per one genome and plasmid of N305); endotoxin levels were measured using the Lonza Chromogenic Endpoint LAL assay; and finally, *S. aureus* host cell proteins (HCP). HCP was measured at various purification steps using several methods to demonstrate the amount of protein reduction. While these methods were not strictly quantitative, the large fold-reduction of values was sufficient to proceed with the *in vivo* studies. Briefly, an area under the curve analysis of the real time ultraviolet readings (280 nm) from anion exchange chromatograms showed a +85% reduction/separation of proteins from fractions used for the study (data not shown). Second, a gradient sodium dodecyl sulfate polyacrylamide gel electrophoresis (SDS-PAGE) of various stages of purification showed several bands present in the mock lysate that were absent in each subsequent step of the purification process. A commercial *S. aureus* HCP enzyme-linked immunosorbent assay (ELISA) quantitation kit was tested (Cygnus F320) but control experiments indicated it did not detect N305. The sterility of individual post-buffer exchanged phage stocks and the final formulation was verified by streaking 20 μL aliquots onto TSA plates and incubating at 37°C for 3 days; plates were checked for colonies daily. Aliquots for injection were prepared under laminar flow conditions and filtered with a 0.2 μm PES membrane.

### Infectious Inoculum Preparation

A -80°C frozen *S. aureus* N305 stock was thawed and grown for 6 h in brain heart infusion (BHI) medium (Oxoid) at 37°C and subcultured in the same conditions overnight. The inoculum was centrifuged for 15 min at 3220 × *g* and washed twice with 10 ml PBS. The number of bacteria was subsequently measured in the inoculum through measurement of OD_600nm_ on a spectrophotometer. Based on these OD results the inoculum was diluted in PBS to the desired number of bacteria. The exact number of CFU of the inoculum was confirmed by plate counts on tryptone soya agar (TSA) plates (Oxoid). An average inoculum dose of 3.45 × 10^2^ CFU/gland was used. All test compounds were injected into the glands at 4 h post-inoculation in a volume of 100 μL.

### Mouse Mastitis Model of Infection

Animal experiments were conducted according to Good Scientific Practice-principles and approved by the Ethical Committee of the Faculty of Veterinary Medicine, Ghent University (approval number EC 2015/127). CD-1 mouse dams (Envigo, Horst, Netherlands) were used 10–14 days after birth of their offspring. The female lactating mice typically weighed 40 g to 50 g at time of use. The pups were removed 1–2 h prior to bacterial inoculation of the mammary glands. A mixture of oxygen and isoflurane (2–3%) was used for inhalational anesthesia of the mice and a bolus of PBS-diluted Vetergesic (i.e., buprenorphine 10 μg/kg, Val d’Hony Verdifarm NV, Belgium) was administered intraperitoneally (i.p.) as analgesic prior to any surgical intervention. A syringe with 32-gauge blunted needle was used to inoculate both L4 (on the left) and R4 (on the right) of the fourth abdominal mammary gland pair with 3.45 × 10^2^ CFU/gland of *S. aureus* (*t* = 0). Each orifice was exposed by a small cut at the near end of the teat and 100 μL of inoculum was injected slowly through the teat canal. After 4 h, the phage cocktail-treated groups received an intramammary dose of 3 × 10^7^ PFU (120 μl). *S. aureus*-infected mice that were treated after 4 h through intramammary injection of 100 μg/gland cefalonium (i.e., a first generation cephalosporin antibiotic used in the dairy industry to treat typically Gram-positive mastitis infections) were also included in the study as a positive control group.

### Clinical and Gross Parameters: Quantifying Bacteria and Phage in Samples from Mammary Glands and Blood

At 24 h post-inoculation (p.i.) with *S. aureus*, mice were sedated i.p. with a ketamine and xylazine mixture prior to sacrifice by cervical dislocation and sampling the injected glands (two per mouse) and blood. Upon sacrifice, the L4 and R4 mammary glands were harvested to quantify both bacteria and phage. Bacterial CFU counts and phage (PFU) counts were obtained after plating serial logarithmic dilutions of mammary gland homogenates, counting colonies or plaques, and transforming counts into base 10 logarithm (log_10_) values. The limits of detection (LOD) were 100 CFU/g of gland and 100 PFU/g of gland, respectively. In addition, blood samples were taken by cardiac puncture from all mice of the uninfected safety control group (only treated with phage cocktail), and plasma was prepared and tested for PFU. Representative images of mammary gland pairs were taken for each group and scored based on clinical and gross parameters by expert veterinarians using a scoring scale from 1 (mild inflammation) to 5 (severe inflammation).

### Histopathological Evaluation of Necrosis and Immune Cell Infiltration

Isolated mammary gland tissue was fixed for 24 h in buffered paraformaldehyde. Representative sections of tissue samples were trimmed and placed in tissue processing cassettes and routinely processed in graded alcohol, cleared in xylene and embedded in paraffin. Paraffin-embedded tissues were sent to Vdx Veterinary Diagnostics (Davis, CA, United States) for histological slide preparation, evaluation and histological scoring. Paraffin blocks for two animals per group were sectioned on a microtome at 4–6 μm, mounted on glass slides and stained with hematoxylin and eosin (H&E) for routine light microscopic evaluation. The pathologist remained blinded to treatment groups during the histopathological examination.

Each sample was evaluated for presence of necrosis, polymorphonuclear neutrophilic granulocyte inflammation (i.e., neutrophilic inflammation), lymphocytic inflammation and bacteria. Each feature was graded semi-quantitatively in ten different high power fields at 200× magnification and an average score for each category was calculated. The semi-quantitative scoring was performed using the following criteria: 0 = *Absent*, the histologic feature (necrosis, neutrophils, lymphocytes, or bacteria) was not observed in the examined 200× field; 1 = *Minimal*, the histologic feature (necrosis, neutrophils, lymphocytes, or bacteria) was present in scant or very small amount in the examined 200× field; 2 = *Mild*, the histologic feature (necrosis, neutrophils, lymphocytes, or bacteria) was consistently present in low numbers through the examined 200× field and normal tissue architecture was maintained; 3 = *Moderate*, the histologic feature (necrosis, neutrophils, lymphocytes, or bacteria) was a prominent and distinctive feature in the examined area (i.e., the majority of glands or tissue in the area exhibited some necrosis, presence of neutrophils, or lymphocytes); 4 = *Severe*, the histologic feature (necrosis, neutrophils, or lymphocytes) was an overwhelming feature of the examined 200× field, in general all glands in the area were affected and normal architecture of the gland was obscured. Quantitative analysis of bacteria is not possible in histological sections.

### Statistical Analysis

Statistical analysis of the *in vivo* data was performed in SPSS and GraphPad Prism, *in vitro* analysis in GraphPad Prism. Because the residuals were not normally distributed, significance was determined with a non-parametric Kruskal–Wallis test followed by Dunns *post hoc* testing for the *in vivo* data, and Mann–Whitney for the *in vitro* and histological data.

## Results

### *In Vitro* Testing of Phage Cocktail Efficacy

**FIGURE 1:**
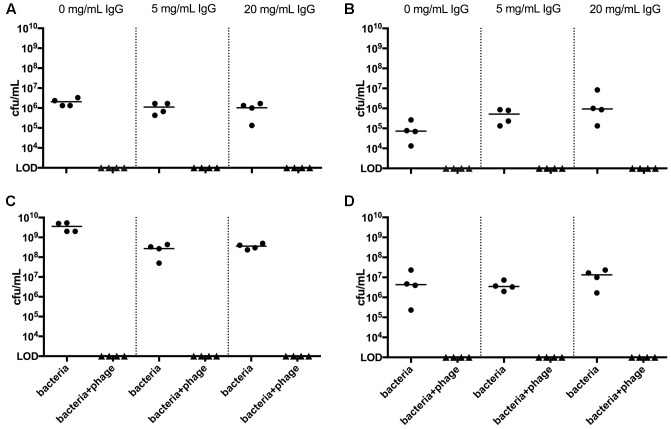
*In vitro* bacteriophage cocktail activity in broth and milk. Bacterial cultures in media **(A,B)** and raw milk **(C,D)** were grown for 4 h, then treated with phage. 3 **(A–C)** and 18 **(B–D)** hours later cultures were plated for CFU. Each circle on the graph corresponds to the CFU/mL value of an individual replicate. Lines represent the median value for each group. Statistically significant differences were observed in all phage-treated cultures, at both time points and regardless of IgG concentration (*P* < 0.05, Mann–Whitney).

The efficacy of a cocktail comprising equal abundances of the two phages was examined *in vitro* using *S. aureus* N305. The phage cocktail was tested both in TSB and in raw milk with the addition of IgG as a potential inhibitor of phage activity after 4 h of bacterial growth (**Figure 1**). Irrespective of the added IgG concentration, the phage cocktail significantly reduced CFU in all treatment cultures (*p* < 0.05) to below the LOD (1 × 10^3^ CFU/mL), demonstrating 3–6 orders of magnitude of killing in broth culture, and 2–4 orders of magnitude in milk. The addition of IgG had no significant impact on CFU in milk or at 4 h in broth (**Figures 1A,C,D**). However, at 18 h in broth (**Figure 1B**) a significant decrease was observed (Mann–Whitney, 0 vs. 5 mg/mL – *P* < 0.05; 0 vs. 20 mg/mL – *P* < 0.05). IgG addition demonstrated no detected impact on phage activity regardless of time or Raw milk in some instances demonstrated background contamination, which was not observed upon phage treatment. Based on these positive results, *in vivo* testing of the phage cocktail was pursued.

### Phage Cocktail Purification

After the phage treatment solution was purified and titered, it was assessed for the presence of bacterial DNA, endotoxin, and *S. aureus* host cell proteins. Each of these parameters were found to be within internally defined acceptable limits, which were based on ascertainable regulatory purity guidelines and published industry standards when available. These values are, per dose: 3 × 10^7^ total PFU, 0.42 EU of endotoxin, and 1.025 ng of host DNA. See “Materials and Methods” section for details of host cell protein testing.

### *In Vivo* Phage Treatment in a Mouse Model of Bovine Staphylococcal Mastitis

#### Colony Forming Units

**FIGURE 2:**
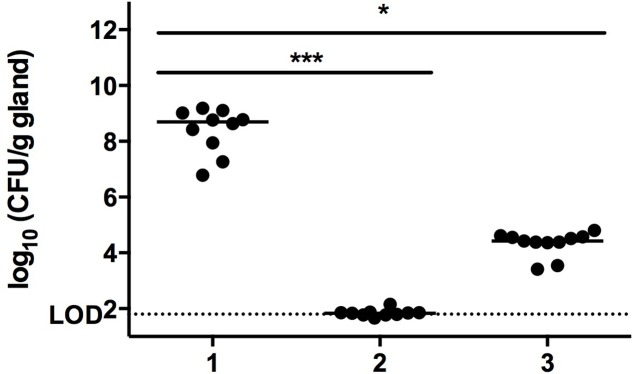
Mammary gland CFU post phage treatment. Twenty-four hours after infection mammary glands were excised, homogenized, and plated in order to count bacterial CFU. Conditions are (1) PBS treatment (negative control group), (2) cefalonium treatment (positive control group), or (3) bacteriophage cocktail treatment (phage treatment group). Each circle on the graph corresponds to the log_10_ CFU/g value of an individual gland. The bar represents the median value for each group. The significant differences between the median values are indicated with (^∗^*P* < 0.05, ^∗∗∗^*P* < 0.001, Kruskal–Wallis) and the dotted line indicates the detection limit (DL).

At 24 h p.i. with *S. aureus*, mammary glands of mice that received PBS (sham, negative control group) had a median bacterial load of 8.70 log_10_ CFU/g of gland which represents an increase of 6.16 log_10_ CFU/g of gland compared to the original inoculum (**Figure 2**). In contrast, mammary glands from mice that received cefalonium (positive control group) had bacterial loads at or below the LOD (**Figure 2**). Mammary glands from mice that received the phage cocktail (phage treatment group) had median bacterial loads of 4.43 log_10_ CFU/g of gland, significantly lower than the negative control group (*p* < 0.001, **Figure 2**).

#### Gross Pathology

**FIGURE 3:**
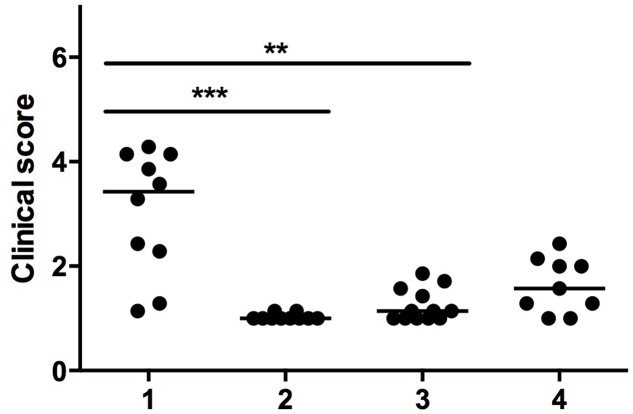
Clinical scoring of mammary glands. Mammary glands were scored from 1 (mild inflammation) to 5 (severe inflammation) by seven independent veterinarians. Each circle on the graph represents one gland, and the bar represents the median value for each group. Conditions are (1) PBS treatment (negative control group), (2) cefalonium treatment (positive control group), (3) bacteriophage cocktail treatment (phage treatment group), and (4) uninfected mice infused with the phage cocktail (safety control group). The significant differences between the median values are indicated with (^∗∗^*P* < 0.01, ^∗∗∗^*P* < 0.001, Kruskal–Wallis).

**FIGURE 4:**
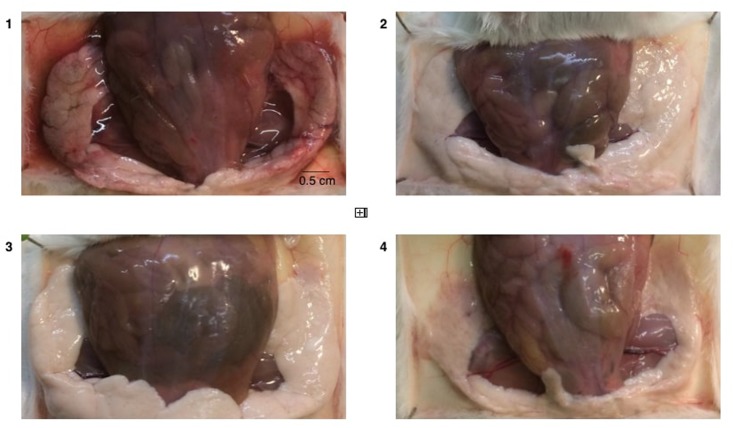
Gross pathology of mammary glands. Representative photographs from dissected mice are shown. Conditions are (1) PBS treatment (negative control group), (2) cefalonium treatment (positive control group), (3) bacteriophage cocktail treatment (phage treatment group), and (4) uninfected mice infused with the phage cocktail (safety control group). Macroscopic differences resulting from the different treatments of the infected mammary glands are clearly visible (e.g., prominent redness and inflammation in 1), and reflected in the scores in **Figure 3**.

Mammary glands were graded for gross pathological appearance (**Figure 3**) which demonstrated significant differences between the negative control group (median score = 3.3), the positive control group (median score = 1.0) and the phage treatment group (median score = 1.1). Mammary glands of the non-infected phage-treated mice had a median score of 1.5 (not significantly different from any other group, *p* > 0.05). Representative post-mortem pictures of mammary glands are shown in **Figure 4**. Mild or lack of inflammation was characterized by the presence of milk in the mammary glands, while redness of the glands indicated a severe local inflammatory reaction accompanied with a slimy exudate, indicative of a high bacterial burden.

#### Descriptive Histopathology

**FIGURE 5:**
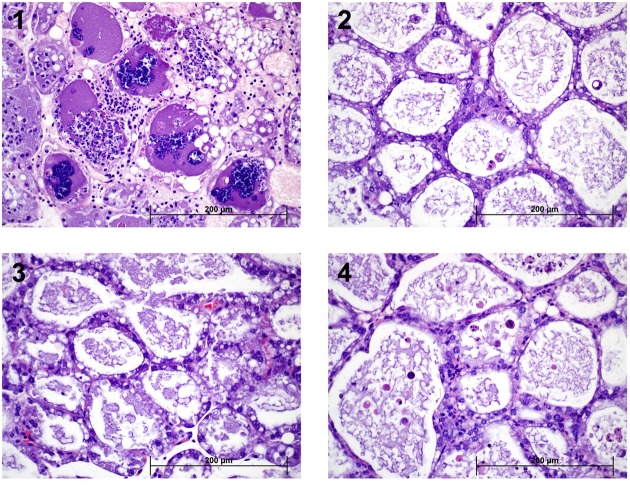
Histopathology. Representative H&E stained tissue sections from each group are shown. (1) Infected plus PBS treatment. Mouse mammary gland. Moderate to severe neutrophilic inflammation and bacterial colonization (dark purple) of mammary glands. The lining epithelium exhibits various stages of degeneration, necrosis and sloughing. (2) Infected plus cefalonium treatment. Healthy epithelial cells lining normal glandular tissue that contains a modest amount of flocculent proteinaceous secretion and rare sloughed necrotic or apoptotic cells. (3) Infected plus phage treatment. This image demonstrates healthy glandular epithelial cells characterized by robust cuboidal shape and a variable degree of clear cytoplasmic vacuolation (lipid). These cells are lining normal glandular tissue that contains a modest amount of flocculent proteinaceous secretion. (4) Uninfected plus phage. This gland is characterized by mild to moderate numbers of sloughed necrotic or apoptotic cells. The intact lining epithelium is otherwise normal.

Representative images after histopathological examination are shown in **Figure 5**. Descriptions of each group follow:

##### Infected plus phosphate-buffered saline (sham) treatment

Representative sections of mammary tissue from animals in this group were characterized predominately by patchy to coalescing intraglandular neutrophilic infiltration with frequent large colonies of coccoid bacteria. The neutrophilic inflammation was often degenerate and intermixed with fragmented nuclear debris. The lining epithelial cells were typically attenuated, discontinuous or otherwise distorted with fading nuclei (karyolysis). Active sloughing of the degenerating epithelial cells into the glandular lumen was frequent. Rounded, hypereosinophilic epithelial cells with pyknotic nuclei were seen to a lesser extent. The glands also contained a variable amount of proteinaceous and lipid-rich secretion.

##### Infected plus phage treatment

Representative sections of mammary tissue from this group were characterized by glands that were consistently lined by robust, cuboidal epithelial cells that exhibited a variable degree of clear cytoplasmic vacuolation. The glandular lumina mostly contained flocculent to slightly globular proteinaceous secretion with modest lipid content. Sloughed, intraglandular epithelial cells that appeared rounded and hypereosinophilic with pyknotic nuclei (interpreted as necrotic or apoptotic) were present in minimal to mild numbers. Patchy, minimal neutrophilic inflammation was recognized only in one gland.

##### Infected plus cefalonium treatment

Representative sections of mammary tissue from this group were lined by epithelial cells that were variably mildly attenuated to often robust with abundant cytoplasm with lipid vacuoles. The luminal contents consisted mostly of flocculent to lipid laden, proteinaceous secretion and included a variable, minimal to moderate, degree of necrotic (or apoptotic) epithelial cells characterized by rounded, hypereosinophilic cytoplasm and pyknotic nuclei. Sparse neutrophilic inflammation was rarely identified. Minimal interstitial lymphocytic inflammation was also identified.

##### Uninfected plus phage treatment

Representative sections of mammary tissue from this group generally contained mild to moderate numbers of sloughed necrotic (or apoptotic) epithelial cells characterized by rounded borders, hypereosinophilic cytoplasm and pyknotic nuclei. The glandular epithelium ranged from attenuated to robust with cuboidal shape with variable degree of cytoplasmic lipid vacuolation. The luminal contents also included flocculent proteinaceous secretion without obvious high lipid content. Only one gland exhibited minimal neutrophilic inflammation. Minimal to mild numbers of interstitial lymphocytes were also identified.

##### Histopathology scoring

**FIGURE 6:**
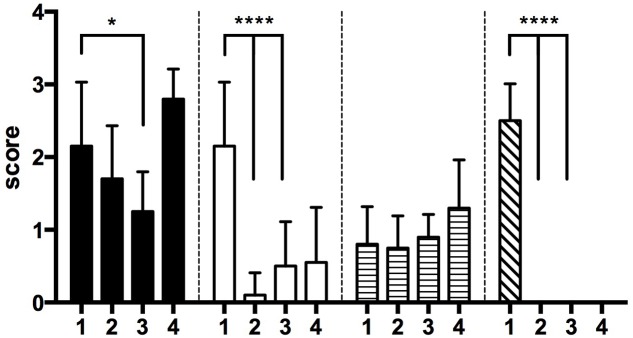
Histological scoring. Tissue sections from excised glands from two mice were processed for histological scoring and read by a veterinary pathologist. Scores (*y*-axis) represent the average of 10 semi-quantitative evaluations for each gland using the following criteria (see Materials and Methods for detailed explanation): 0 = *Absent*, 1 = *Minimal*, 2 = *Mild*, 3 = *Moderate*, 4 = *Severe*. Groups consist of: black bars = Necrosis, white bars = PMN, horizontal striped bars = Lymphocytes, and diagonal striped bars = Bacteria. Conditions are (1) PBS treatment (negative control group), (2) cefalonium treatment (positive control group), or (3) bacteriophage cocktail treatment (phage treatment group), and (4) uninfected mice infused with the phage cocktail (safety control group). Statistically significant differences were observed between PBS treatment (1) and cefalonium (2) or PBS treatment (1) and phage treatment (3) where indicated (^∗^*P* < 0.05, ^∗∗∗∗^*P* < 0001, Mann–Whitney). Error bars represent standard deviation.

Sectioned and stained tissues were blindly scored on a semi-quantitative scale (**Figure 6**). Infected PBS-treated mammary glands (group 1) showed moderate necrosis (black bars), had the highest neutrophilic inflammation (white bars) and highest bacteria scores (diagonal bars). Infected cefalonium treated mammary glands (group 2) did not display a significant decrease in necrosis or lymphocytes compared to the infected PBS-treated glands, but were lower in neutrophil numbers as well as bacteria. Infected phage treated mammary glands (group 3) showed the lowest scores for necrosis, and unlike the infected cefalonium treated glands these were significantly lower than the infected PBS- treated glands. They also had significantly lower neutrophilic scores, mild to moderate lymphocytic inflammation (horizontal bars) and did not have observable bacteria. As expected, the uninfected phage treated mammary glands (group 4) had low neutrophilic, lymphocytic and bacterial scores, similar to those observed for both the cefalonium and the phage treated infected groups, however, they had the highest necrosis score. All groups had minimal and similar lymphocytic scores.

#### Clinical Observations

Clinical behavior was also affected in the infected PBS-treated mice. These mice were less motile and were grouped together in the cage, in marked difference to all other treatment groups, which the mice were very alert and active. As motility can be depressed by hypothermia, core body temperature of the mice was measured before sacrifice. Infected PBS-treated mice had the lowest average core body temperature (35°C), while those from the phage treated infected group had the highest average core body temperature (36.1°C), however, this latter observation did not reach significance (*P* > 0.05). This indicates that all mice were sacrificed before reaching their humane endpoints.

#### Plaque Forming Units

**FIGURE 7:**
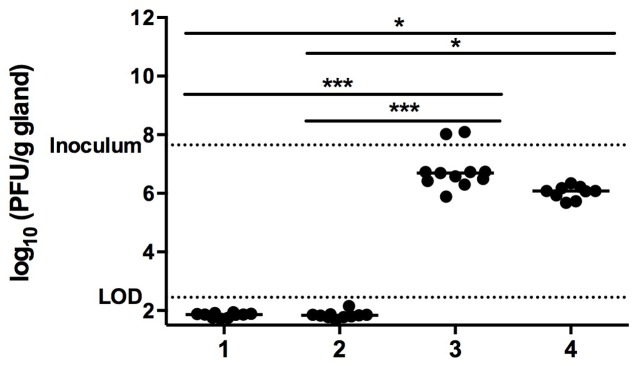
Mammary gland PFU post phage treatment. After *Staphylococcus aureus* infection and treatment mammary glands were excised, homogenized, and plated on bacterial lawns in soft agar for PFU determination. Conditions are (1) PBS treatment (negative control group), (2) cefalonium treatment (positive control group), or (3) bacteriophage cocktail treatment (phage treatment group), and (4) uninfected mice infused with the bacteriophage cocktail (safety control group). Each circle on the graph corresponds to the log_10_ PFU/g value of an individual gland. The bar represents the median value for each group. The significant differences between the median values are indicated with ^∗^*P* < 0.05, ^∗∗∗^*P* < 0.001, Kruskal–Wallis). Dotted lines represent the limit of detection (LOD) and the phage inoculum dose (inoculum).

At 24 h p.i. no phages were detected in the mammary glands of either the PBS treated or the cefalonium treated infected groups (**Figure 7**). In phage treated mice mammary glands either infected or uninfected had similar PFU. The PFU of all phage-treated groups indicate a reduction of the original bacterial inoculum, with a median value (log_10_ PFU/g of gland) of infected plus phage treatment of 6.69, and a median value of uninfected plus cocktail of 6.08, vs. the inoculum value of 7.6. (**Figure 7**). Interestingly, the infected plus phage group PFU counts were slightly higher relative to those of the uninfected plus phage group (i.e., 6.69 vs. 6.08). Although this difference was not statistically significant, it suggests that the phages productively infected their *S. aureus* host and were likely amplified. Regarding systemic spread, LOD of 2.45 log10 PFU/g gland.

## Discussion

The current study investigated the potential of phage therapy for the treatment of bovine staphylococcal mastitis, by combining complementary *in vitro* and *in vivo* strategies. Following the *in vitro* characterization and selection of a promising bacteriophage cocktail, a preclinical mouse model for bovine mastitis was used to study the *in vivo* effects of this candidate therapy against *S. aureus*.

It was first demonstrated *in vitro* that *S. aureus* was reduced to below the detection limit by the phage cocktail both in media as well as in raw milk, regardless of IgG concentration. This latter is a key observation as it differs from the results of several previous studies (Das and Marshall, 1967; O’Flaherty et al., 2005a; Gill et al., 2006b; Tanji et al., 2015) which reported a either a lack of or a significant reduction of phage activity in raw milk or its components. These authors attributed this observation to either whey protein binding of the bacterial cells or bacterial aggregation, purportedly driven by IgG lowering the adsorption rate of phage (Tanji et al., 2015). Although bacterial aggregation was not directly examined as a part of this study, CFU counts suggest that it did not generally occur, as increases in IgG concentration would lead to fewer CFU if aggregation took place. This was true, with the exception of TSB at 18 h. The lack of aggregation in our study is in marked contrast to the three previously cited papers and may be attributable to strain-level variability in aggregation potential. Biofilm and aggregate formation are complex and multifactorial processes in *S. aureus*, and variability between strains has been observed by us (data not shown) and others (Haaber et al., 2012; Thammavongsa et al., 2015). In the Tanji report, a different bovine mastitis isolate was used, SA003 (NBRC 110649) (Tanji et al., 2015), and O’Flaherty used the *S. aureus* strains DPC5246 and DPC5645 from the Dairy Products Research Centre culture collection (O’Flaherty et al., 2005a). Thus it is possible that the strain used in this study, N305, does not aggregate to the same degree as these other strains. However, N305 was also used in a study in which whey proteins were demonstrated to inhibit phage activity (Gill et al., 2006b). Although the specific cause or causes of these different results is unknown, it may at least partly be explained by factors such as the use of different phages, the variability inherent in raw milk, and differences in experimental design.

The preceding *in vitro* bacteriophage characterization was performed to successfully select therapeutic phage candidates, prompting further investigation to correlate *in vitro* phage performance with *in vivo* efficacy against bovine *S. aureus*. Quantitative bacterial loads, histological evaluation, and qualitative clinical and gross macroscopic changes all strongly indicated that treatment with the phage cocktail significantly decreased the intramammary bacterial loads and inflammation caused by the bovine mastitis isolate *S. aureus* N305. Moreover, the quantitative PFU counts strongly indicated that the phage treatment was able to maintain high local phage titers in the murine mammary gland (**Figure 7**) without spreading systemically.

Of particular interest is the improvement in clinical outcome in phage-treated mice, as measured by gross pathology and clinical ante-mortem observation, despite the failure of phage to achieve microbiological cure as observed with cefalonium treatment (**Figure 2**). The gross appearance of the tissues, along with the behavior and appearance of the mice, closely matched the cefalonium treated group. Phage are known to live in symbiosis with bacteria and follow a predator/prey PK/PD (pharmacokinetic/pharmacodynamic) model (Lenski and Levin, 1985; Drulis-Kawa et al., 2012), meaning that depending on the conditions phage alone may not completely eliminate microbial populations in the way small molecule antibiotics do. The clinical benefit may lay in the ability to reduce pathogen burden to the point that the host immune system can resolve remaining infection, a benefit observed in phage trials that demonstrate microbiological cure (Kutter et al., 2010; Abedon et al., 2011). Had the our experiments been carried out beyond 24 h it is possible that further reduction in CFU would have similarly been observed, due to the immune response as well as due to continued activity of phage infection. However, additional time can increase the risk of the development of bacterial resistance. Although microbiological cure is ultimately necessary in a *S. aureus* mastitis treatment, early clinical improvement may still allow a more rapid return to production.

Last but not least, phage treatment of uninfected glands appeared to be safe and well-tolerated. Treatment did not induce significant inflammation by gross pathological observation, and histological evaluation did not reveal significant differences between either neutrophilic or lymphocytic inflammation. Phage treatment is also considered as generally safe for humans, which has been confirmed numerous times in patients from the Phage Therapy Unit at Hirszfeld Institute (Miedzybrodzki et al., 2009). In humans, phage therapy has been recently reported as being both safe and successful (Schooley et al., 2017) and was reviewed by Kutter et al. (2010) and Schooley et al. (2017). Moreover, it has been suggested that the application of phage preparations may probably influence and diminish the inflammatory reaction that accompanies bacterial infection in humans as well (Górski et al., 2016). However, the necrosis score for this group was the highest of the four groups. This appears paradoxical, as the infected and phage treated group encouragingly had a significantly lower necrosis score. Although the necrosis scores were similar between infected PBS-treated and uninfected phage treated, the histopathology of the two lesions were qualitatively different. Moreover, the mechanism underlying this observed necrosis is unclear. A recent publication on the histopathology of *S. aureus* mastitis states that necrosis can be induced by neutrophil influx migration (Chinchali and Kaliwal, 2014), yet at the 24-h time point this phenomenon was not observed histopathologically.

The most likely explanation relates to the involution that takes place upon weaning of the pups prior to inoculation in the mouse mammary gland infection model. Involution safeguards the host from extensive inflammation in the post-lactation period (Watson, 2006). Normal involution typically causes very rapid and massive cell death by apoptosis, hence the rounded, hypereosinophilic cells with pyknotic nuclei are more likely cells undergoing this natural apoptosis, rather than necrosis. The histomorphology of cellular necrosis and apoptosis may not always be distinctively different. Further, an accurate interpretation of histological necrosis often requires a broader context than is provided in a blinded evaluation. In the context of this study, high “necrosis” scores without associated gross confirmation or accompanying histological inflammation can more likely be attributed to controlled or programmed cellular death (i.e., apoptosis) rather than cellular death due to disease, injury or ischemia (i.e., necrosis). Because these intracellular Gram-positive bacteria are typically the most difficult to eradicate from the (bovine) mammary gland with standard antibiotic treatment, this intriguing phenomenon warrants further investigation. Finally, the same authors state that “tissue damage can initially be caused by bacteria and their products.” Purification to remove contaminants and quality testing was performed on the phage treatment solution, however, it is conceivable that a low local contaminant level, such as endotoxin or a *S. aureus* toxin, sufficient to induce necrosis remained.

Overall, the phage-only inoculated group demonstrated no gross clinical symptoms or observable macro- or microscopic deleterious findings, consistent with data from two previous studies that treated 6–8 weeks old mice with either phage K (Gill et al., 2006a) or phage ϕMR11 (Matsuzaki et al., 2003) by intraperitoneal injection, although both these purification methods differed from the one used herein. Of interest, the phage K study, in which dairy cattle received intramammary infusions of phage K, reported an increase in somatic cell count (Gill et al., 2006a). This is interesting in light of the current findings, as it is feasible that the increase in cell count was due to apoptotic cells rather than an inflammatory response as we postulate herein. It is also feasible that it could be caused by an immune response to contaminants in the treatment, or to the phages themselves. In contrast, another similar bovine study in which two phages were intramammarily infused reported no such response (O’Flaherty et al., 2005b).

## Conclusion

The combined *in vitro* and *in vivo* results of the current study indicate that phage therapy is a promising alternative to antibiotics for the treatment of bovine staphylococcal mastitis. Although this innovative strategy was independently proposed by three other groups, those studies date from more than a decade ago (Matsuzaki et al., 2003; O’Flaherty et al., 2005b; Gill et al., 2006a), and the bacteriophages were hampered by bovine whey proteins inhibiting their interaction with *S. aureus*. The latter was not an issue with our novel bacteriophage cocktail. Besides a direct antibacterial effect, a synergistic effect with the classical antibiotics can also be envisaged, potentially allowing a lower dose and/or shorter treatment with these drugs. Future research will focus on validating this pilot study in an inoculum dose-titration study to determine the optimal phage dose for *S. aureus* treatment and clinical improvement, and provide the basis for further translation of our proof-of-concept in dairy cattle. Future directions may include iterating this process to establish a relationship between individual phage characteristics and *in vivo* performance to refine the phage scoring metrics so as to enable predictive, high-throughput selection of good candidate phages for future therapeutic cocktails.

## Author Contributions

KB helped design and perform *in vivo* experiments, analyzed *in vivo* results, prepared graphs, performed statistics, and helped write the manuscript. RH designed experiments, analyzed data, wrote and edited the manuscript, and developed figures. ZH developed phage purification and manufacturing strategies, chromatography experiments, quality control assays and cocktail formulation, and wrote part of the manuscript. MR performed *in vitro* analysis of phages. MŻ performed *in vitro* experiments in broth and milk, wrote part of the manuscript, and analyzed results and prepared graphs. TS prepared samples and performed histopathological analyses. JS helped perform *in vivo* experiments, analyzed *in vivo* results, prepared graphs, and helped write the manuscript. RL helped develop and perform initial *in vitro* experiments in broth and milk. AK analyzed phage genomes. BM oversaw the development, characterization and preparation of the phage cocktail. EM developed *in vivo* protocol and oversaw performance of *in vivo* experiments and data analysis; helped write and edit the manuscript. LM defined study objectives and roles/responsibilities of all parties, approved budget, and executed collaboration contract from EpiBiome. Developed protocol and coordinated collaboration logistics and study execution, and helped write the manuscript.

## Conflict of Interest Statement

The authors declare that the research was conducted in the absence of any commercial or financial relationships that could be construed as a potential conflict of interest.
